# Hemangioma of Tongue With Multiple Phleboliths Involving Multiple Spaces of the Right Side of the Head and Neck Region: A Case Report Along With the Importance of Specialized Imaging

**DOI:** 10.7759/cureus.44330

**Published:** 2023-08-29

**Authors:** Bhuvaneshwari M, Priya Ramani, Bharath TKS, Aravind Warrier S, Sreedevi Jeyakumaran

**Affiliations:** 1 Department of Oral Medicine and Radiology, Thai Moogambigai Dental College and Hospital, Chennai, IND; 2 Department of Oral Medicine and Radiology, Sri Ramachandra Institute of Higher Education and Research, Chennai, IND

**Keywords:** diascopy, mri, ct, phleboliths, hemangioma

## Abstract

Hemangioma is one of the most common tumors of dilated blood vessels, which is usually present at birth and involutes over time. Although considered the most common tumor in the head and neck region, the oral cavity is less commonly affected. The occurrence of hemangioma in the tongue is very rare (14%). Changes in blood flow are dynamic within the hemangioma resulting in thrombus and phleboliths. Phleboliths are small blood clots that occur in a vein, which usually hardens over time due to calcifications. The phleboliths are also called vein stones, which tend to be oval-shaped and are generally less than 5 millimeters in diameter. This paper reports a case of hemangioma of the tongue. On routine radiographic investigation (orthopantomogram), multiple phleboliths were found extending over the right side of the jaw involving the ramus and body of the mandible, which was an incidental finding. On specialized imaging, the extent of the phleboliths turned out to involve multiple spaces, which was unexpected.

## Introduction

Hemangiomas are true neoplasms of the head and neck (50%), the occurrence of hemangioma in the tongue is very rare (14%). They are seen within a few weeks of birth, which rapidly grows and involute over a period [1]. Hemangiomas are characterized by an increase in turnover and proliferation of endothelial cells [2]. Mulliken and Glowacki, in 1982, classified vascular deformities into two major groups: hemangioma and vascular malformations [3]. Hemangiomas based on their histological appearance are further sub-classified as capillary hemangioma and cavernous hemangioma. Seven percent (7%) of all benign soft-tissue tumors consist of hemangioma. Females show a higher prevalence than males (3:1) [4]. The sites involved are the lip (63% of cases), tongue (14% of cases), buccal mucosa (14% of cases), gingiva, palatal mucosa, salivary glands, alveolar ridge, and jaw bones [3,5].

Hemangiomas may induce phlebolith formation due to changes in blood flow. Calcified thrombi found in veins, venulae, and sinusoidal vessels are phleboliths, especially of cavernous type hemangiomas. A mixture of calcium carbonate and calcium phosphate salts comprises phleboliths. They also occur in normal tissue in spite of normal serum calcium and phosphate levels. Usually, they are varied and of multiple sizes and they are randomly distributed [2]. A few head and neck hemangioma with phleboliths cases are documented, among which some cases were in the buccal mucosa, 10 cases in the parotid, submandibular, and sublingual glands, one case each was reported in the floor of the mouth, hypopharynx, base of the tongue, and parapharyngeal space, and four in the masseter [6]. Phleboliths are diagnosed by standard radiograph, which is an important diagnostic tool, whereas CT, MRI, and ultrasound play an additional important role. CT is useful in the detection of phleboliths and MRI is useful in the detection of vascular lesions [2].

## Case presentation

A 40-year-old female patient reported to the Oral Medicine and Radiology department, with a chief complaint of pain in her lower right back tooth region for the past three days. History of presenting illness revealed pain was sudden, sharp, aggravated on mastication of food, and relived on medication. The patient was healthy and her medical history was not relevant. The patient had no deleterious habits. The patient also gave a history of growth in her tongue, which was present since childhood.

**Figure 1:**
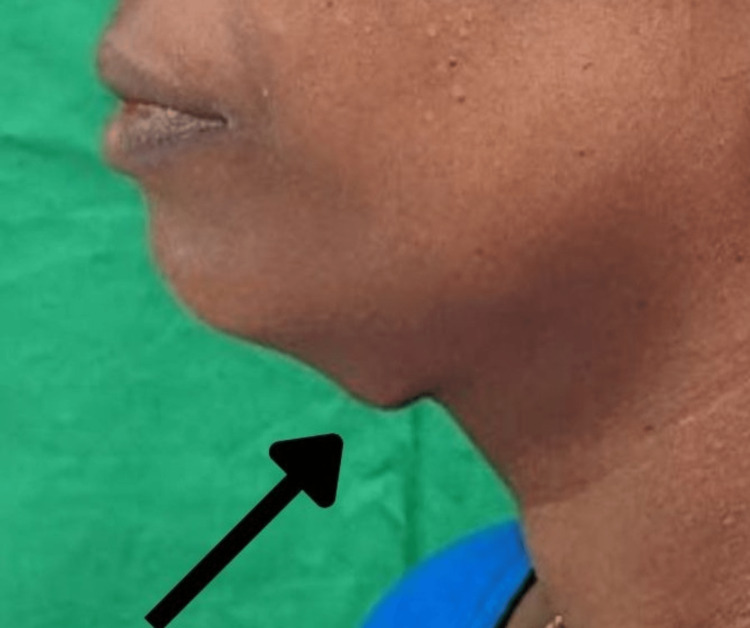
Lateral view of the patient’s profile showing a single round swelling seen in the submental region

On extra-oral examination, a single well-defined round swelling was evident on the submental region approximately measuring about 1.5x1.5 in diameter. The skin over the swelling appeared normal with no evidence of color change. On palpation, all the inspected findings were confirmed. It was nontender, soft to firm in consistency, and freely movable in all directions, the skin over the swelling was pinchable, and the swelling was compressible but not reducible (Figure 1).

**Figure 2:**
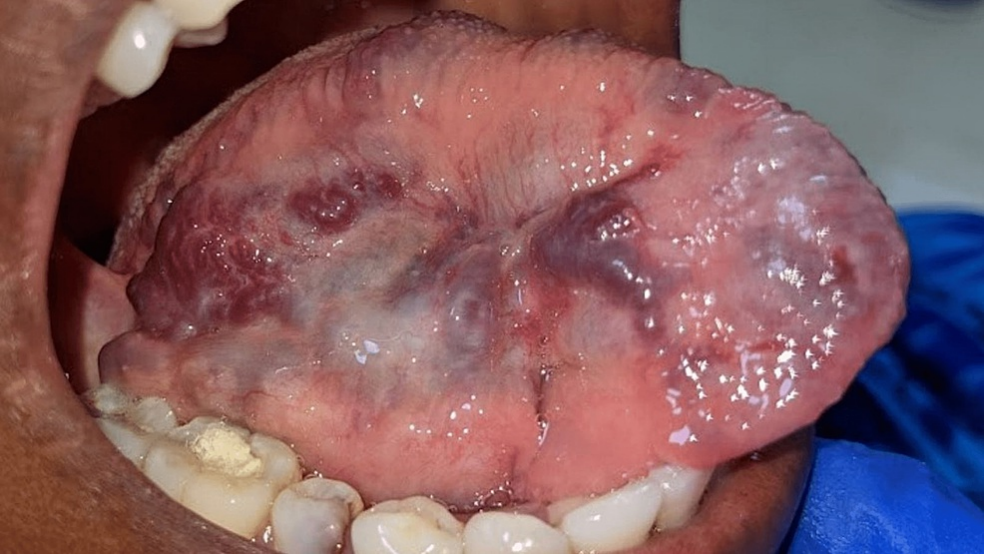
Dorso-ventral surface of the right side of the tongue showing multiple, discrete, purplish-blue swellings measuring about 5X3 cm approximately

**Figure 3:**
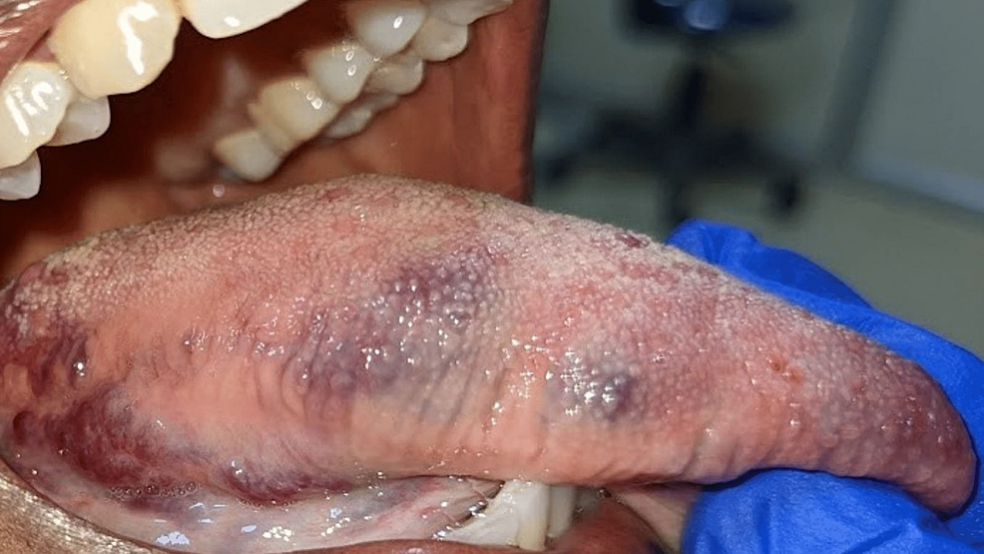
Lateral border of the right side of the tongue showing three discrete purplish-blue swellings approximately measuring about 1X1 cm

On intra-oral examination, dental caries was seen in 45 and there was partially erupted 48. Subsequently, multiple discrete swellings were noted on the dorsoventral aspect of the tongue, which were unilateral. The swelling roughly measured about 5x3 cm; and was purplish blue in color with no evidence of any ulceration. The surface of the swelling was irregular. On palpation, all the inspected findings were confirmed. The swelling was non-tender and soft in consistency, with no pulsation, bruits, or thrills heard (Figures 2, 3).

**Figure 4:**
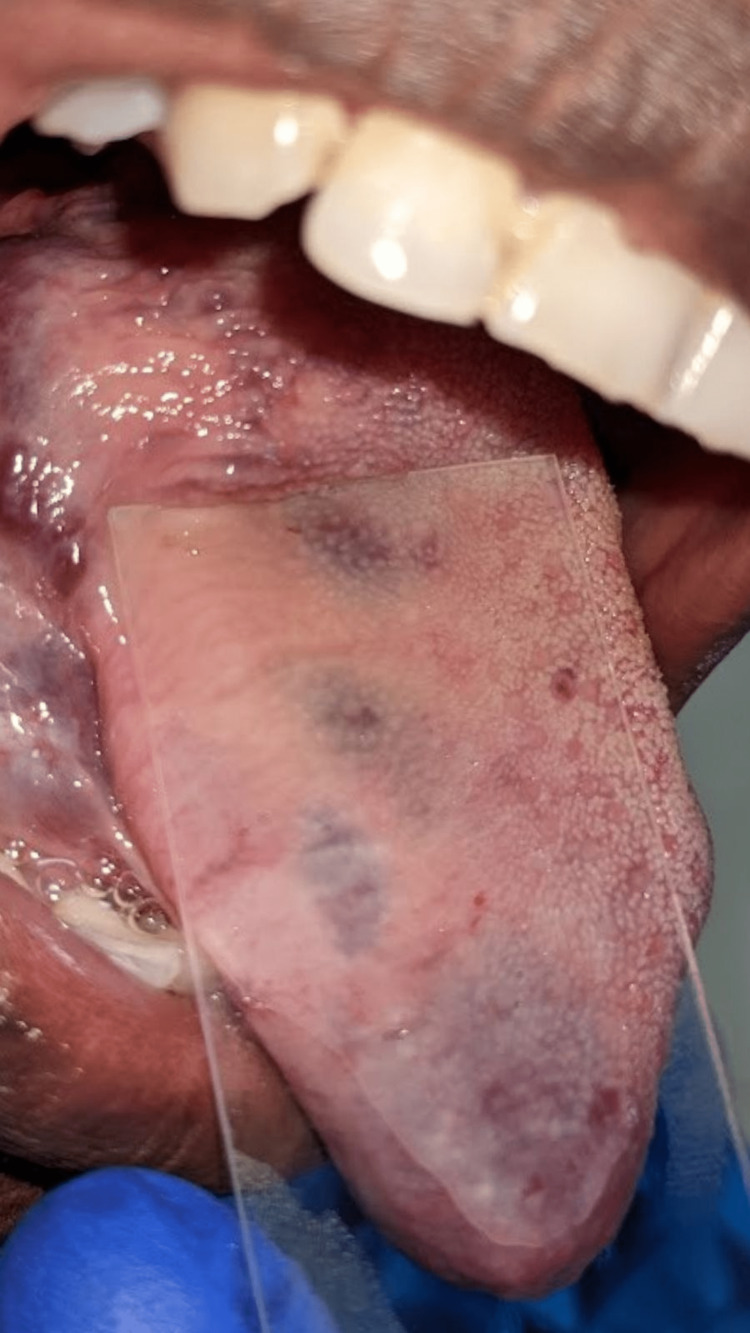
Diascopy positive on the lateral border of the tongue

**Figure 5:**
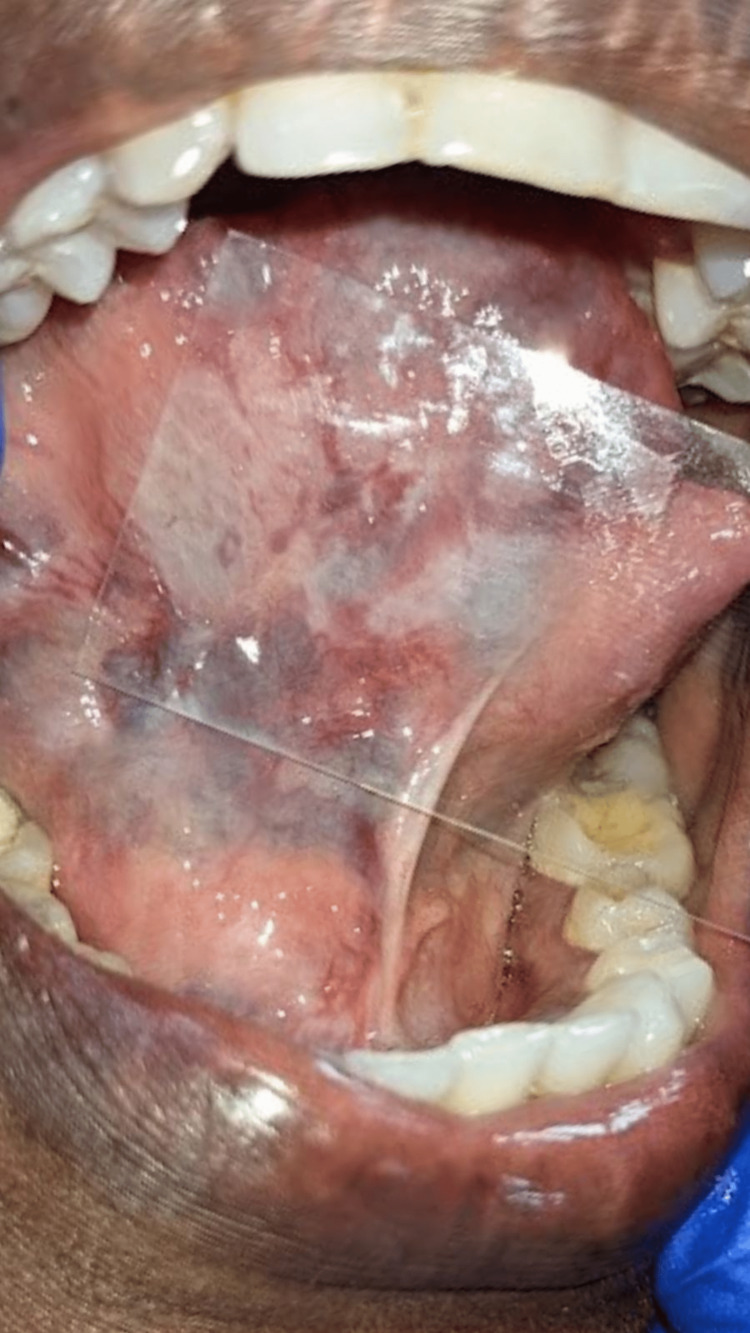
Diascopy positive on the ventral surface of the tongue

**Figure 6:**
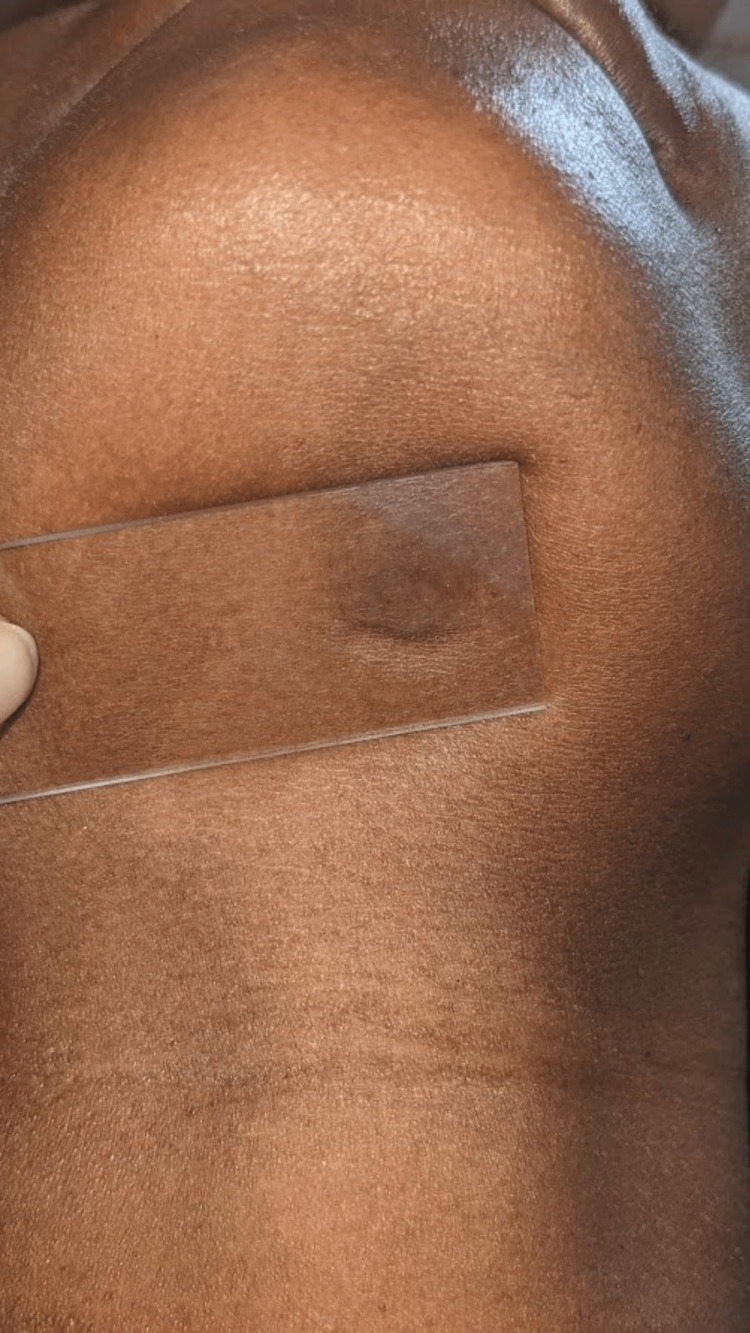
Diascopy negative on the swelling present in the submental region

**Figure 7:**
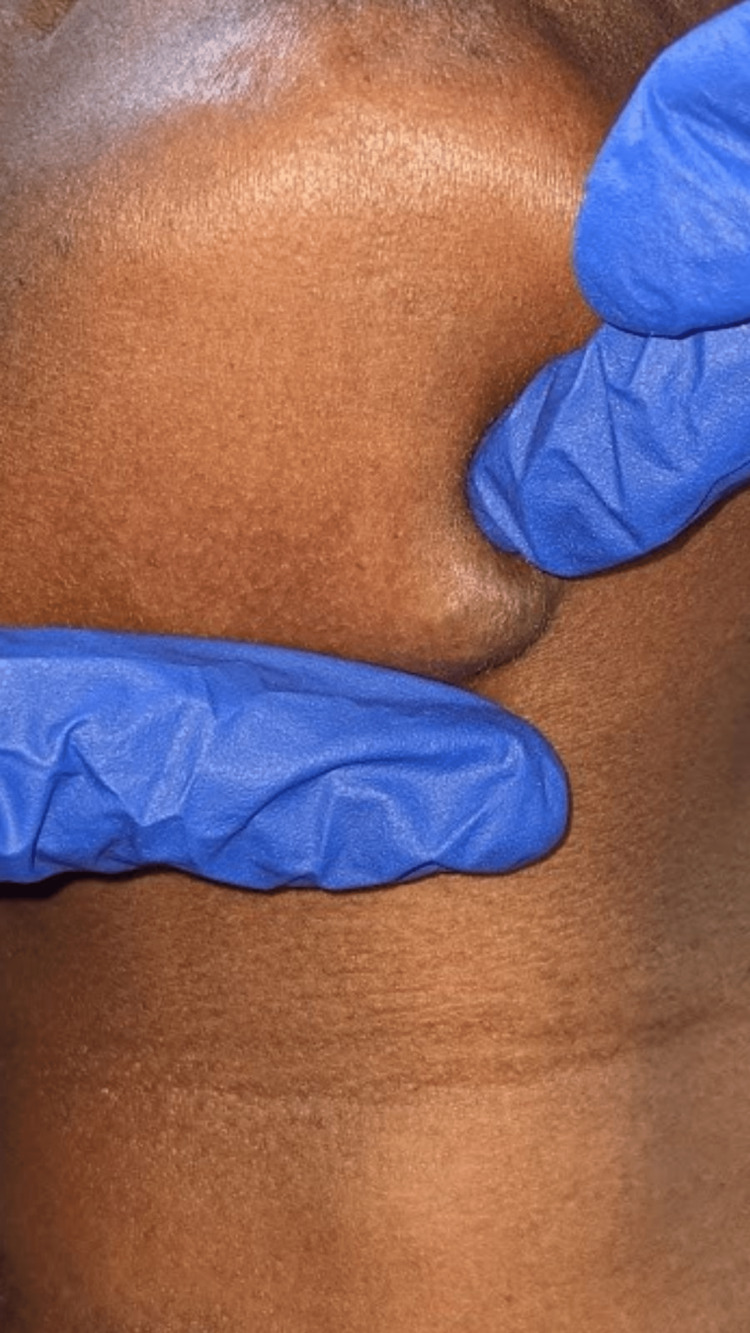
Slip test positive on the swelling present in the submental region

As a chairside investigation, diascopy was performed on the swelling of the tongue, which turned out to be positive (Figures 4, 5) and negative in the submental region (Figure 6). A slip test was performed in the submental region, which was positive (Figure 7). Based on the chief complaint, a provisional diagnosis of dental caries in 45 and partially erupted tooth in relation to 48 was given. Based on the clinical appearance of the swelling and positive diascopy a diagnosis of hemangioma of the tongue was given. A diagnosis of lipoma in the submental region was given based on the positive slip test.

**Figure 8:**
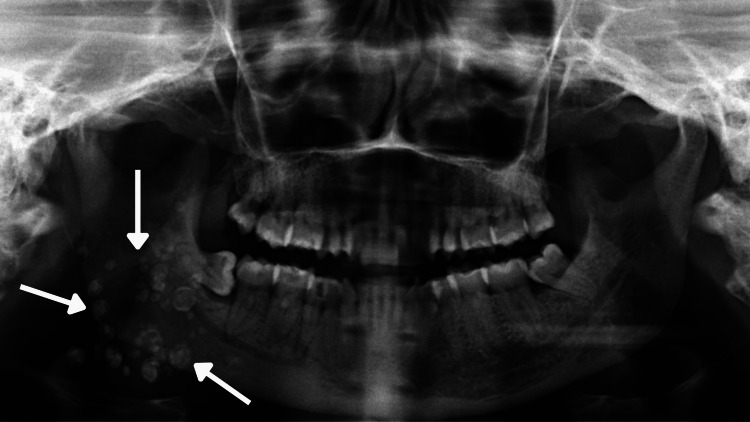
OPG reveals multiple radio-opaque structures in the right side of the mandible, which was suggestive of multiple phleboliths OPG: orthopantomogram

Based on the chief complaint, the patient was subjected to an orthopantomogram (OPG). OPG in relation to 45 revealed radiolucency involving the enamel, dentin, and pulp, with a widening of the periodontal ligament space, and loss of lamina dura suggestive of dental caries with apical periodontitis. OPG in relation to 48 revealed the tooth distal to 47 was partially embedded within the bone and the long axis of 48 was perpendicular to the long axis of 47, suggestive of a horizontally impacted 48. To our surprise, multiple radio-opaque structures were seen on the right side of the mandible, which were suggestive of multiple phleboliths (Figure 8).

**Figure 9:**
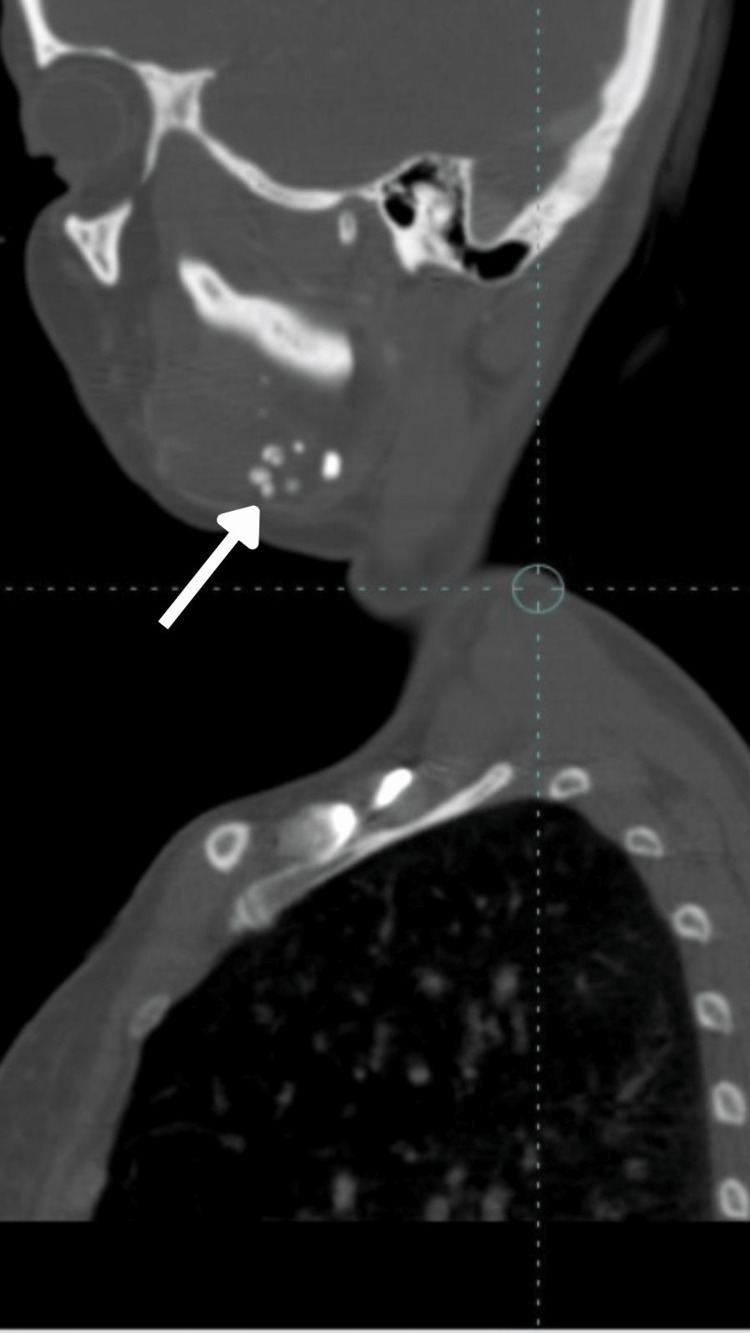
Sagittal CT image with contrast showing multiple phleboliths extending to the submandibular space

**Figure 10:**
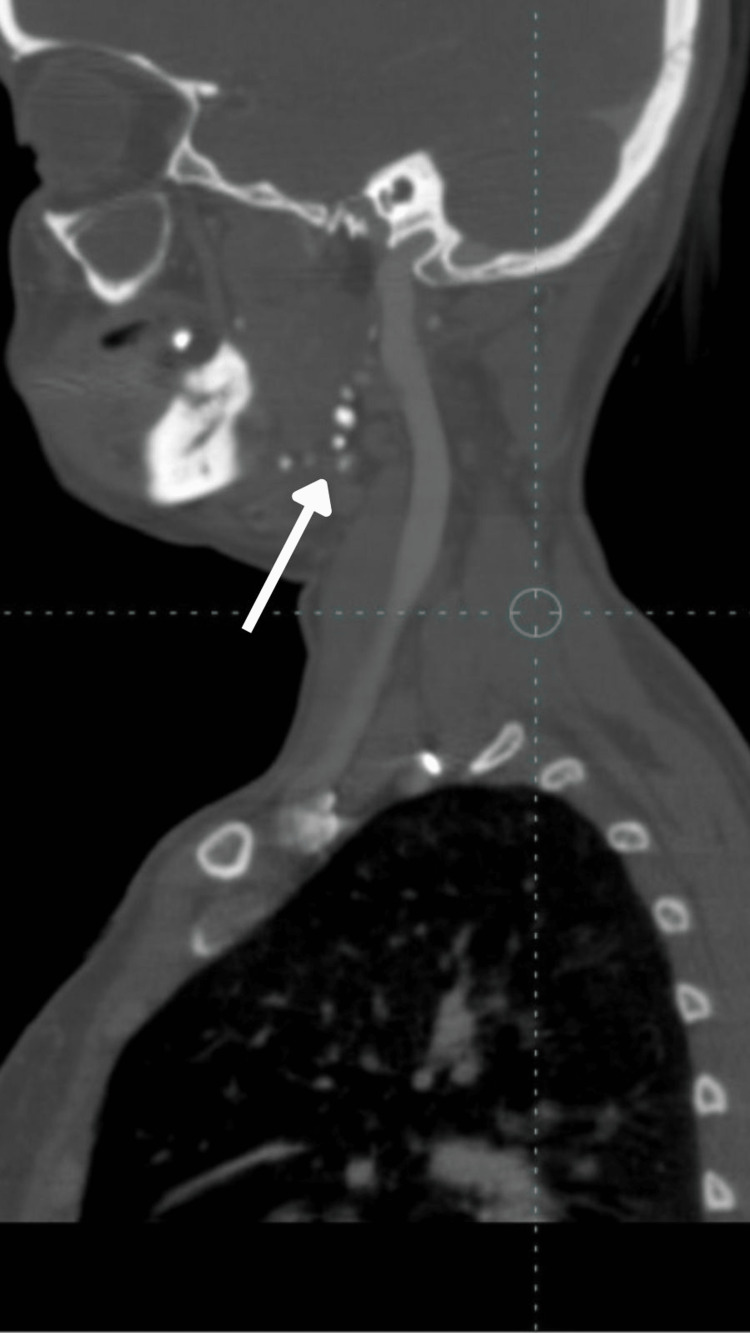
Sagittal CT image with contrast showing multiple phleboliths extending to the oral pharyngeal region

**Figure 11:**
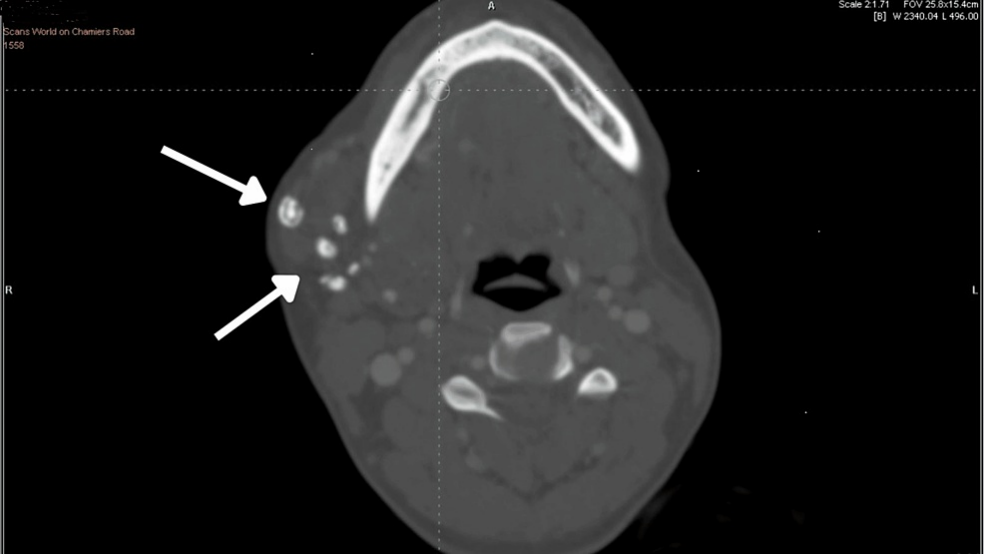
Axial CT image with contrast showing multiple phleboliths extending over the right parotid space and right temporomandibular joint

**Figure 12:**
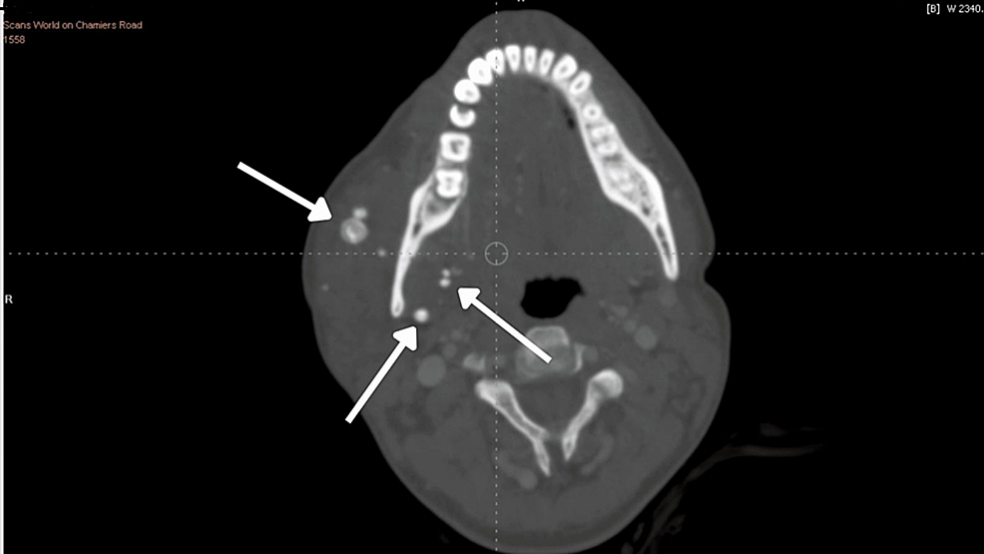
Axial CT image with contrast showing multiple phleboliths extending over the masseteric space and the lateral wall of the pharyngeal region

Further, the patient was subjected to CT (computed tomography) plain and CT contrast along with MRI (magnetic resonance imaging). The report revealed a large, ill-defined, multiloculated STIR (short tau inversion recovery) hyperintense area with septation and lobulated margins extending over the right side of the tongue, right sublingual region, and floor of the mouth suggestive of hemangioma. Multiple phleboliths extending into the masseteric space (involving the masseter and pterygoid), right temporomandibular joint, submandibular space extending to pharyngeal space, right side of the soft palate, right pharyngeal wall (nasopharyngeal, oropharyngeal), right parotid space involving the parotid gland (Figures 9-12).

The lesion exhibits diffuse heterogenous enhancement on contrast study with prominent vascular supply from enlarged branches of the right external carotid artery. The lesion measured 8.8 cm (craniocaudally) x 6.1 cm (in width) x 10.1 cm (anteroposteriorly).

**Figure 13:**
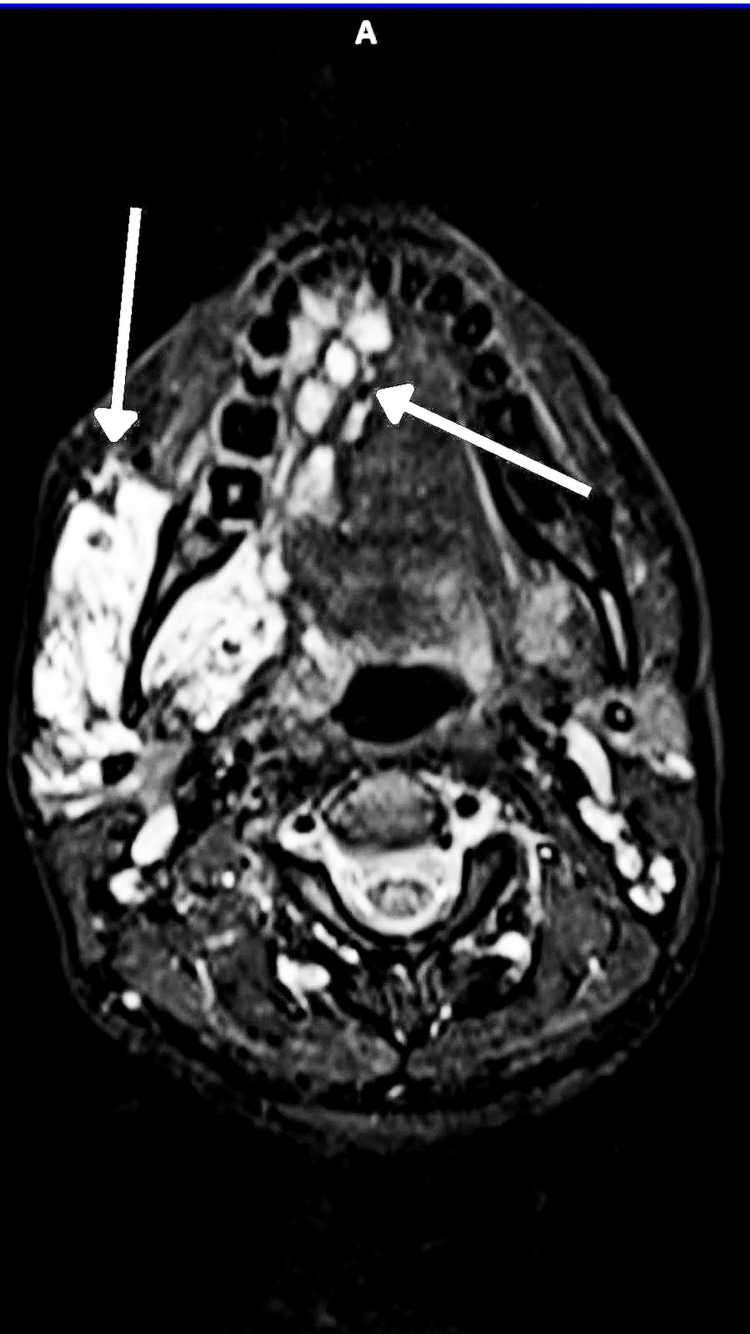
Axial MRI image showing a well-defined STIR hyperintense region extending over the lateral glossoepiglottic fold, adjacent epiglottis, and lateral pharyngeal wall with extension along the aryepiglottic fold on the left side STIR: short tau inversion recovery

**Figure 14:**
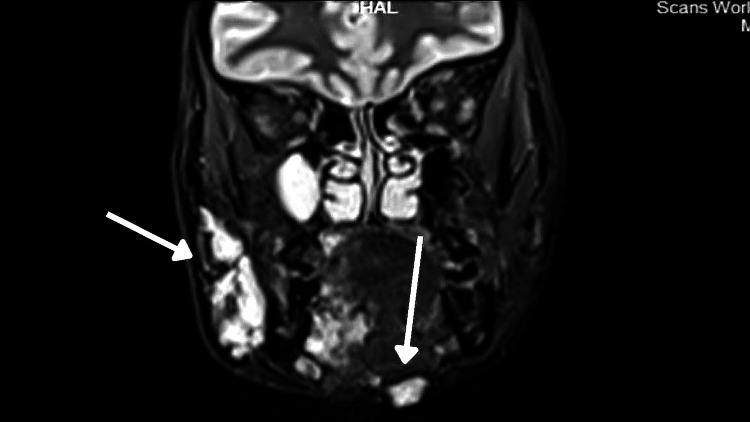
Coronal MRI image showing small discrete STIR hyperintense foci in the subcutaneous plane of the submental region to the left side of the midline STIR: short tau inversion recovery

There was a small discrete STIR hyperintense foci in the subcutaneous plane of the submental region to the left side of the midline. It was a well-defined STIR hyperintense region extending over the lateral glossoepiglottic fold, adjacent epiglottis, and the lateral pharyngeal wall with extension along the aryepiglottic fold on the left side, likely a small hemangioma (Figures 13, 14).

## Discussion

The term hemangioma consists of the Greek words ‘haema,’ which means blood; ‘angio,’ which means vessel, and ‘oma,’ meaning tumor [4,6]. Hemangioma is classified depending upon the diameter of the blood vessel as the capillary or cavernous type. Strawberry hemangioma is another name for the capillary type, which is composed of small walled vessels of capillary size that are lined by a single layer of flattened or plump endothelial cells and surrounded by a discontinuous layer of pericytes and reticular fibers. The cavernous type contains large blood-filled spaces called cavities that are separated by a scanty connective tissue stroma. The size of the hemangioma varies from pinpoint to several cm in diameter, and the color of the lesion ranges from bright red to purple, depending upon the position. The surface appears like a flat, raised, or nodular mass. They usually do not metastasize but rather proliferate or involute with time [4].

Initially, phleboliths associated with vascular anomalies were found in the splenic vein by Canstatt in 1843. A characteristic property of venous or cavernous hemangioma is phleboliths, which are calcified nodules. These originate due to vessel wall injury or result from stagnation of the flow of blood, which results in damage to the intima. Healing involves the formation of a protective thrombus that may calcify as a part of the healing process. Thrombus formation is favored by slowing with stagnation of blood flow. In turn, the thrombus organizes with laminar fibrosis and leads to a progression of central necrosis. Crystallization by deposition of calcium, phosphate, and apatite, and with calcification of thrombus, a phlebolith evolves [6].

Specialized imaging techniques play a major role in the diagnosis of phleboliths. In the present case, it was an incidental finding when we acquired an orthopantomograph. Round to oval radio-opaque structures were randomly distributed, and varying sizes were seen on the right side of the ramus and body of the mandible. Differential diagnoses for head and neck phleboliths can be sialoliths, tonsilloliths, calcified lymph nodes, foreign bodies, carotid atherosclerotic plaques, cysticercosis, healed acne lesions, and miliary osteoma cutis [7].

Sialoliths usually appear to be present in a single line, whereas phleboliths are randomly distributed and usually co-exist with hemangioma. Because of certain disadvantages of the panoramic radiograph, which is a two-dimensional image causing superimposition, magnification, and distortion, an additional three-dimensional imaging modality is definitely needed like CT, CT contrast, MRI, ultrasound, and cone beam computed tomography (CBCT).

In this case, a specialized radiograph, such as CT plain, CT contrast, or MRI, is requested to have a detailed three-dimensional examination of the site and extent of the calcified structures. On 2-D imagining it revealed radiopaque mass present only in the right side of the mandible but on 3-D imaging, it revealed its presence extending over the right side of the tongue, right sublingual region, floor of the mouth, masseteric space (involving the masseter and pterygoid), right temporomandibular joint, submandibular space extending to the pharyngeal space, right side of the soft palate, right pharyngeal wall (nasopharyngeal, oral pharyngeal), and right parotid space involving the parotid gland. The extent of the hemangioma can only be determined using a CT scan or MRI but its detectability on MR images is slightly superior to that on CT images. Phleboliths detectability on CT images is superior to that on MR images [8]. To our surprise, the provisional diagnosis of lipoma in the submental region turned out to be a small hemangioma on MRI, where again specialized imaging modality played a role.

## Conclusions

In the case of an oral hemangioma, it is important to undergo a radiographic investigation to rule out the presence of phleboliths. The diagnosis of hemangioma is important to rule out other vascular lesions. Though 2-D imaging reveals the presence of phleboliths, 3-D imaging is definitely needed to find the exact involvement and extent of the lesion. This plays a vital role in proper treatment planning and management of the patient. This patient was asymptomatic and not surgically managed because of the involvement of phlebolith over multiple spaces.
